# Association of interleukin-10 promoter genetic variants with T-cell and B-cell activation in HIV-1 infection

**DOI:** 10.1186/1742-4690-9-S2-P168

**Published:** 2012-09-13

**Authors:** D Naicker, B Julg, C McClurg, M Ghebremichael, F Porichis, J Zupkosky, M Jaggernath, M Mokgoro, P Goulder, B Walker, D Kaufmann, T Ndung'u

**Affiliations:** 1 HIV Pathogenesis Programme, Durban, South Africa; 2 Ragon Institute of MGH, MIT and Harvard, Boston, MA, USA; 3 University of Oxford, Department of Paediatrics, UK

## Background

Interleukin-10 (IL-10) is a potent immunoregulatory cytokine, with promoter polymorphisms that have previously been associated with HIV-1 susceptibility and pathogenesis. Association of IL-10 SNPs with markers of CD4, CD8 and B cell activation has not previously been investigated.

## Methods

Two IL-10 polymorphisms were genotyped by TaqMan allelic discrimination and markers of activation of CD4, CD8 and B cells were measured in 63 individuals using flow cytometry. The following monoclonal antibody combinations were used: anti-CD3 Pac-blue, anti-CD38 PE-Cy7, anti-HLA-DR ACP-Cy7, anti-CD95 PE, anti-CD19 Alexa-700, anti-IgG PE-Cy5, anti-PD-1 APC, anti-Ki67 FITC, anti-CD4 Qdot605 and anti-CD8 Qdot655.

## Results

Previous studies on this cohort showed a significant association between -1082GG and higher median IL-10 expression, compared to the -1082AA/AG (p= 0.0006). The -592AA and -1082AA (both previously shown to be associated with low-IL-10 production) had significantly higher median expression of HLA-DR on CD4 and CD8 cells respectively, compared to the other genotypes (-592AA vs. -592CA p= 0.005, -592AA vs. -592CC p= 0.03 and -1082AA vs. -1082AG p= 0.02). The -592AA genotype also had a higher median expression of CD95 and PD-1 on CD4 cells (-592AA vs. -592CA p= 0.0227, -592AA vs. -592CC p= 0.0270 and -592AA vs. -592CA p= 0.01 respectively). The -592CC and -1082GG genotypes associated with higher median expression of IgG on the surface of B cells (-592CC vs. -592AA p= 0.0207 and -1082GG vs. -1082AG p= 0.0392, -1082GG vs. -1082AA p= 0.0051).

## Conclusion

These data suggest that IL-10 polymorphisms that affect cytokine production and HIV pathogenesis may affect markers of immune activation and exhaustion in response to antigen, and suggest a beneficial role for IL-10 in chronic HIV infection. Further studies on association between IL-10 and the quality and magnitude of immune responses in HIV infection are needed.

